# Involvement of cAMP-guanine nucleotide exchange factor II in hippocampal long-term depression and behavioral flexibility

**DOI:** 10.1186/s13041-015-0130-1

**Published:** 2015-06-24

**Authors:** Kyungmin Lee, Yuki Kobayashi, Hyunhyo Seo, Ji-Hye Kwak, Akira Masuda, Chae-Seok Lim, Hye-Ryeon Lee, SukJae Joshua Kang, Pojeong Park, Su-Eon Sim, Naomi Kogo, Hiroaki Kawasaki, Bong-Kiun Kaang, Shigeyoshi Itohara

**Affiliations:** Behavioral Neural Circuitry and Physiology Laboratory, Department of Anatomy, Brain Science & Engineering Institute, Kyungpook National University Graduate School of Medicine, 2-101, Dongin-dong, Jung-gu, Daegu, 700-842 Korea; Laboratory for Behavioral Genetics, RIKEN Brain Science Institute, 2-1, Hirosawa, Wako-shi, Saitama, 351-0198 Japan; Department of Psychiatry, Faculty of Medicine, Fukuoka University, 7-45-1, Nanakuma, Jonan-Ku, Fukuoka, 814-0180 Japan; Department of Biological Sciences, College of Natural Sciences, Seoul National University, 599 Gwanangno, Gwanak-gu, Seoul, 151-747 Korea; Department of Brain and Cognitive Sciences, College of Natural Sciences, Seoul National University, Seoul, 151-746 Korea

**Keywords:** Synaptic plasticity, Spatial memory, Reversal learning, Knockout mice

## Abstract

**Background:**

Guanine nucleotide exchange factors (GEFs) activate small GTPases that are involved in several cellular functions. cAMP-guanine nucleotide exchange factor II (cAMP-GEF II) acts as a target for cAMP independently of protein kinase A (PKA) and functions as a GEF for Rap1 and Rap2. Although cAMP-GEF II is expressed abundantly in several brain areas including the cortex, striatum, and hippocampus, its specific function and possible role in hippocampal synaptic plasticity and cognitive processes remain elusive. Here, we investigated how cAMP-GEF II affects synaptic function and animal behavior using cAMP-GEF II knockout mice.

**Results:**

We found that deletion of cAMP-GEF II induced moderate decrease in long-term potentiation, although this decrease was not statistically significant. On the other hand, it produced a significant and clear impairment in NMDA receptor-dependent long-term depression at the Schaffer collateral-CA1 synapses of hippocampus, while microscopic morphology, basal synaptic transmission, and depotentiation were normal. Behavioral testing using the Morris water maze and automated IntelliCage system showed that cAMP-GEF II deficient mice had moderately reduced behavioral flexibility in spatial learning and memory.

**Conclusions:**

We concluded that cAMP-GEF II plays a key role in hippocampal functions including behavioral flexibility in reversal learning and in mechanisms underlying induction of long-term depression.

## Background

The newly identified cAMP-binding proteins known as cAMP-guanine nucleotide exchange factors (cAMP-GEFs) have provided novel insights regarding the action of cAMP on intracellular signaling and cellular functions. cAMP-GEFs, which are directly activated by cAMP, are GEFs responsible for the activation of the Ras-related small GTPases Rap1 and Rap2 [1, 2]. Previous pharmacological studies have demonstrated that cAMP-GEFs play a role in increasing neurotransmitter release and inducing synaptic potentiation in cortical and hippocampal pyramidal neurons [3–5]. In addition to their presynaptic functions, pharmacological studies have also shown that cAMP-GEFs can control the extracellular signal-regulated kinase (ERK) and p38 mitogen-activated protein kinase (MAPK) pathways through the activation of Rap proteins, and modulate synaptic plasticity via α-amino-3-hydroxy-5-methyl-4-isoxazloe propionic acid (AMPA) receptor trafficking in postsynaptic densities [6]. cAMP-GEFs are subdivided in cAMP-GEF I (also known as RapGEF3 or Epac1) and II (also known as RapGEF4 or Epac2), and these two isoforms show differential expression in the brain. cAMP-GEF I is expressed broadly at low levels in the adult brain, whereas cAMP-GEF II is strongly expressed in the mature brain, with high levels in the cerebral cortex and CA3 and dentate gyrus of the hippocampus [2].

cAMP-GEF II has been implicated in various brain functions such as memory and sociability. In mice, knockdown of cAMP-GEF II reduced fear memory retrieval in contextual fear conditioning [7], and cAMP-GEF II deficiency impaired social and communication behavior [8]. Furthermore, a recent work has shown that cAMP-GEF I/II double-null mice on a 129sv background presented deficits in hippocampal spatial learning, with impairment of long-term potentiation (LTP), but not long-term depression (LTD) [9]. However, the specific role of cAMP-GEF II in hippocampal synaptic plasticity and cognitive functions as well as their related mechanisms remain elusive. In the present study, we investigated whether cAMP-GEF II contributes to the modulation of hippocampal Schaffer collateral (SC)-CA1 synapses, and how cAMP-GEF II is involved in hippocampus-dependent cognitive functions, using a cAMP-GEF II knockout mouse generated on a C57BL/6 J background.

## Results

### Generation of *cAMP-GEF II*^*−/−*^ mice and cAMP-GEF II protein expression in the brain

**Fig. 1:**
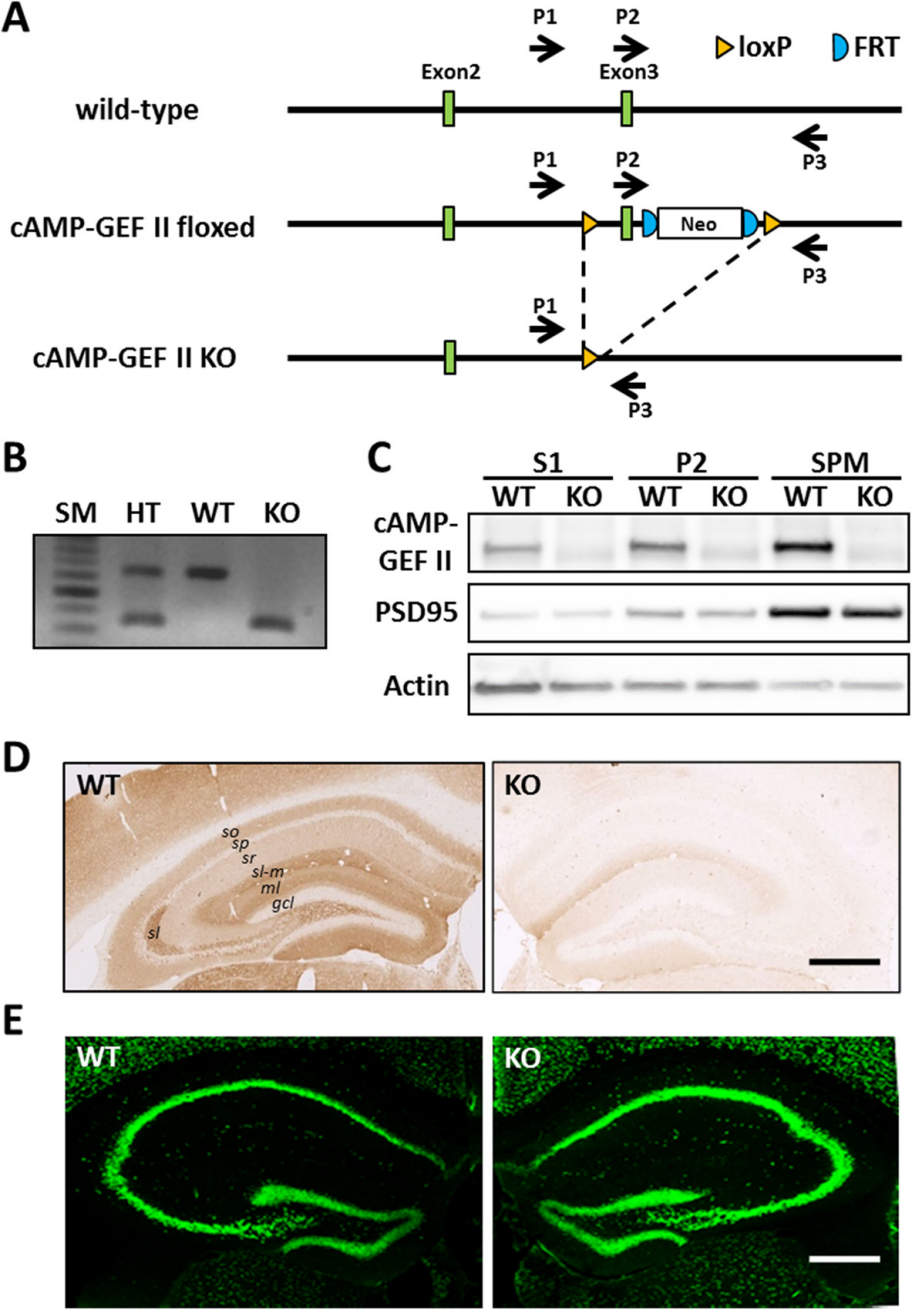
Characterization of *cAMP-GEF II* ^*−/−*^ mice. **a.** Schematic diagram for wild-type, floxed, and knockout (KO) alleles of cAMP-GEF II. Floxed mice were generated by gene targeting using MS12 ES cells derived from the B6 strain, and KO mice were generated by expressing Cre recombinase in the germ cells of the floxed mice (arrow, locus of primer (P1, P2, and P3) for genomic PCR). **b**, Genomic PCR analysis of *cAMP-GEF II* gene deletion in *cAMP-GEF II* ^*+/−*^ (HT, heterozygous), *cAMP-GEF II* ^*+/+*^ (WT, wild-type), and *cAMP-GEF II* ^*−/−*^ (KO, knockout) mice. **c**, Western blot analysis of cAMP-GEF II protein expression in fractionated brain lysates. cAMP-GEF II protein expression was compared among S1 (postnuclear), P2 (crude membrane), and SPM (synaptic plasma membrane) fractions. cAMP-GEF II protein was highly expressed in SPM fractions, which also presented high expression of PSD95. Note that cAMP-GEFs protein expression was completely abolished in the brain of *cAMP-GEF II* ^*−/−*^ mice. **d**, Immunohistochemical analysis of cAMP-GEF II expression in brain tissue sections. Strong immunolabeling was observed in the cortex and hippocampus of WT mice, but was absent in KO mice. In the hippocampus, immunoreactivity for cAMP-GEF II was relatively low in the *stratum pyramidale (sp)* of the *Cornu Ammonis* (CA) as well as in the *granular cell layer (gcl)* of the dentate gyrus; while the *stratum oriens (so), radiatum (sr),* and *lacunosum moleculare (sl-m)*, as well as the *molecular layer (ml)* of the dentate gyrus showed strong immunoreactivity for cAMP-GEF II. **e**, Immunofluorescence for NeuN showed that there was no difference in morphology of the hippocampus between the two genotypes. Scale bars = 500 μm in D, E. Abbreviations: SM, size marker

We confirmed first the disruption of the *cAMP-GEF II* gene in *cAMP-GEF II*^*−/−*^ mice by genomic PCR using tail tissues (Fig. 1a and b), and assessed expression of cAMP-GEF II protein by western blotting using fractionated brain tissues of wild-type and *cAMP-GEF II*^*−/−*^ mice, before assessing the physiological functions of cAMP-GEF II in the hippocampus. We observed that cAMP-GEF II protein was expressed in wild-type mice, but abolished in *cAMP-GEF II*^*−/−*^ mice (Fig. 1c). Moreover, western blot analysis revealed prominent expression or reduction of cAMP-GEF II in the synaptic plasma membrane (SPM) fraction of wild-type and *cAMP-GEF II*^*−/−*^ mice, respectively (Fig. 1c), suggesting that cAMP-GEF II protein is mainly expressed at the postsynaptic membrane and postsynaptic density (PSD). In addition, we confirmed that cAMP-GEF II was highly expressed in dendritic processes (i.e., the *stratum oriens, radiatum, lacunosum moleculare*, and *lucidum* of the CA, as well as in the molecular layer of the dentate gyrus), rather than in cellular layers of hippocampus (i.e., *stratum pyramidale* of the CA and granular layer of the dentate gyrus) of wild-type mice, while its expression was completely abolished in the hippocampus of *cAMP-GEF II*^*−/−*^ mice (Fig. 1d). Finally, there were no morphological anomalies in the hippocampus (Fig. 1e), or other brain areas (data not shown) of *cAMP-GEF II*^*−/−*^ mice compared to wild-type mice.

### Long-term potentiation is moderately decreased in *cAMP-GEF II*^*−/−*^*mice*

**Fig. 2:**
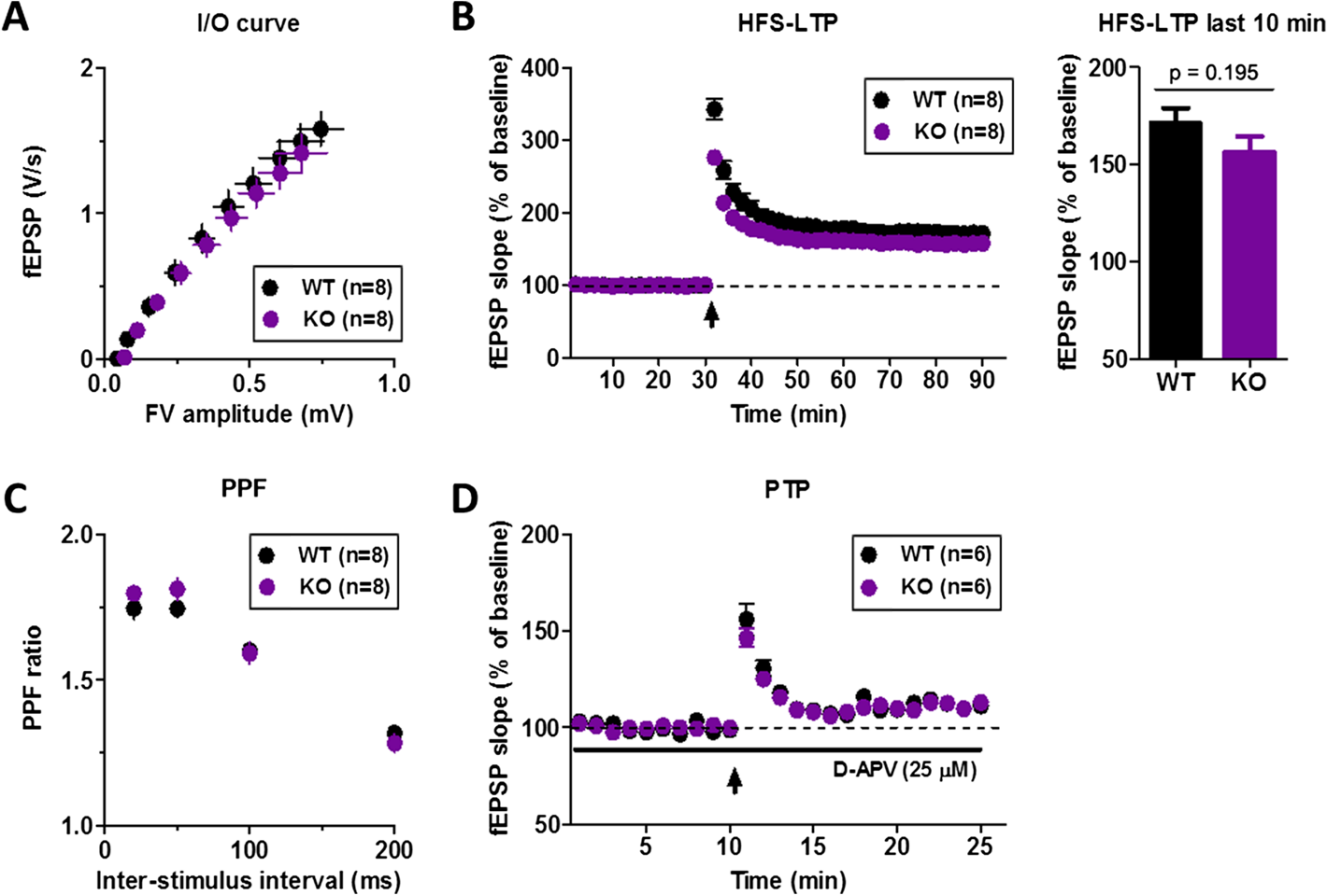
Basal synaptic properties and long-term potentiation in wild-type and *cAMP-GEF II* ^*−/−*^ mice. **a**, Input–output curves as a measure of baseline excitatory synaptic transmission showed no difference between the two genotypes (WT = 8 slices from six mice; KO = 8 slices from six mice). **b**, Long-term potentiation (LTP) induced by high frequency stimulation (arrow, 1x HFS; 100 Hz for 1 s) was slightly impaired without statistical significance in Schaffer collateral-CA1 (SC-CA1) synapses of *cAMP-GEF II* ^*−/−*^ mice (WT = 171.54 ± 7.61 %, 8 slices from eight mice; KO = 156.74 ± 7.76 %, 8 slices from eight mice; unpaired t-test, *p* = 0.195). **c**, Paired pulse facilitation (PPF) ratio did not differ between wild-type and *cAMP-GEF II* ^*−/−*^ mice (WT = 8 slices from six mice; KO = 8 slices from six mice). **d**, Post-tetanic potentiation (PTP) also did not differ between wild-type and *cAMP-GEF II* ^*−/−*^ mice (WT = 8 slices from six mice; KO = 8 slices from five mice; arrow, 1x HFS). Abbreviations: fEPSP, field excitatory postsynaptic potential; HFS, high frequency stimulation

We tested the input–output function in SC-CA1 synapses of hippocampal slices in order to evaluate the effect of the lack of cAMP-GEF II on basal synaptic transmission using extracellular field potential recording. Basal synaptic strength was indistinguishable between the two genotypes (Fig. 2a), indicating that genetic deletion of cAMP-GEF II did not affect basal synaptic transmission. We then examined the physiological role of cAMP-GEF II in hippocampal synaptic plasticity. High frequency stimulation (HFS, 100 Hz for 1 s) of afferent fibers induced LTP in SC-CA1 excitatory synapses in both wild-type and *cAMP-GEF II*^*−/−*^ mice (Fig. 2b). However, the magnitude of LTP during the last 10 minutes in *cAMP-GEF II*^*−/−*^ mice was moderately smaller than in wild-type mice (WT: 171.54 ± 7.61 % of baseline, n = 8 slices from eight animals; *cAMP-GEF II*^*−/−*^: 156.74 ± 7.76 % of baseline, n = 8 slices from eight animals; *p* = 0.195) (Fig. 2b), although there was no statistical significance.

### Presynaptic functions are intact in *cAMP-GEF II*^*−/−*^*mice*

Considering previous reports showing that cAMP-GEF activation by 8-(4-chlorophenylthio)-2’-*O*-methyl-cAMP (8-CPT-cAMP), a selective cAMP-GEFs agonist, induced enhancement of neurotransmitter release in cultured hippocampal neurons [4], a deficiency in cAMP-GEF II can change the neurotransmitter release at presynaptic terminals, leading to changes in synaptic plasticity. To further explore the presynaptic involvement of cAMP-GEF II in hippocampal synapses, we monitored two forms of presynaptic short-term plasticity: paired-pulse facilitation (PPF) and post-tetanic potentiation (PTP). PPF was induced by stimulation of a pair of SC-CA1 synapses at short intervals (20, 50, 100, or 200 ms), which is known to be sensitive to presynaptic release probability [10]. PTP was analyzed using a protocol composed of a single train of tetanic stimulation (100 Hz for 1 s) in the presence of D(−)-2-amino-5-phosphonovaleric acid (D-APV; 25 μΜ) to block NMDA receptor-dependent postsynaptic modifications. Both PPF and PTP were indistinguishable between wild-type and *cAMP-GEF II*^*−/−*^ mice (Fig. 2c, d, respectively), suggesting that hippocampal presynaptic functions associated with short-term plasticity were unchanged in *cAMP-GEF II*^*−/−*^ mice.

### NMDA receptor-dependent long-term depression (NMDAR-LTD) is impaired in *cAMP-GEF II*^*−/−*^ mice

**Fig. 3:**
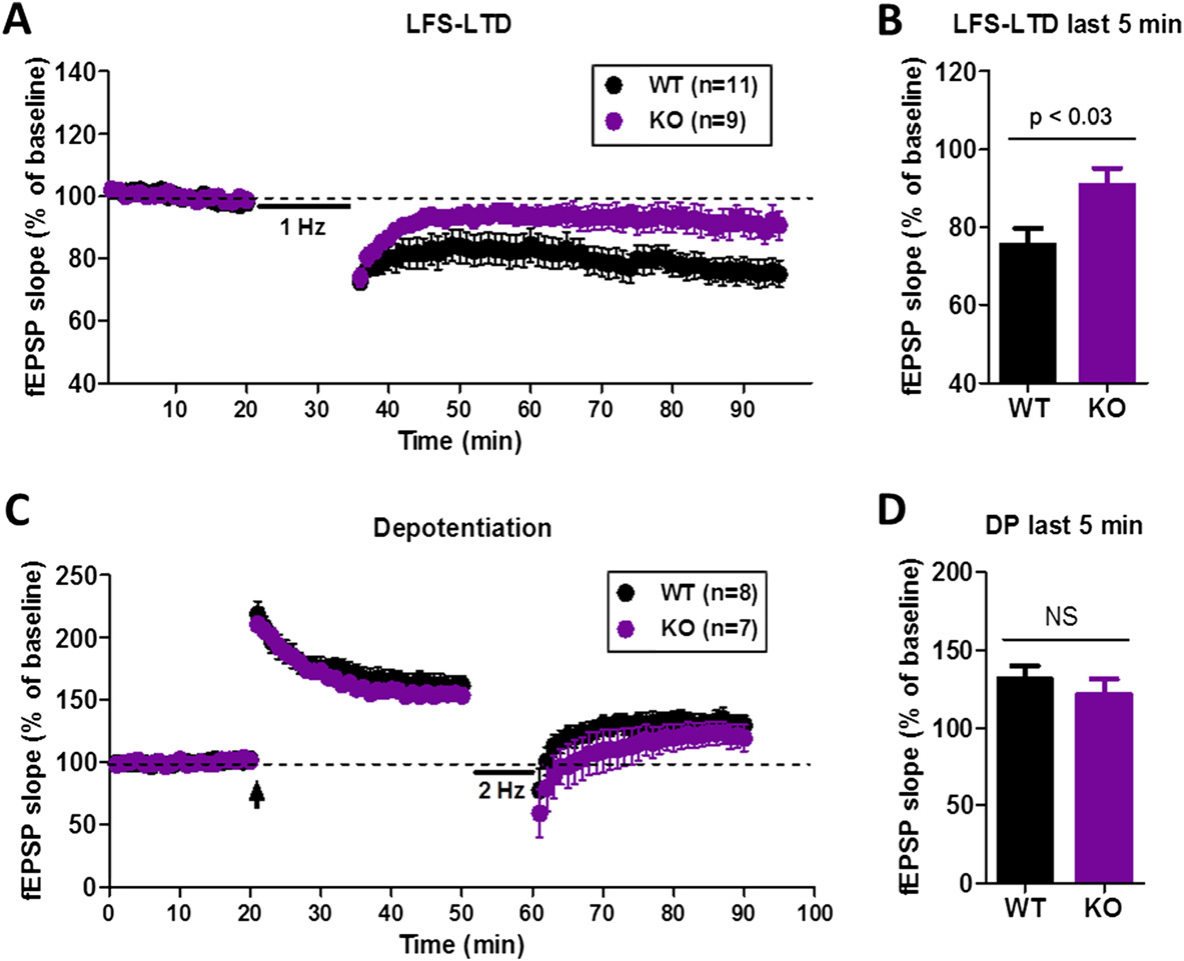
NMDA receptor-mediated long-term depression and depotentiation in wild-type and *cAMP-GEF II* ^*−/−*^ mice. **a**, Long-term depression (LTD) induced by low frequency stimulation (1x LFS; 1 Hz for 15 min) was impaired in *cAMP-GEF II* ^*−/−*^ (KO) mice. **b**, There was a significant difference in NMDA receptor-LTD between wild-type and *cAMP-GEF II* ^*−/−*^ mice during the last 5 min of recording (WT = 75.54 ± 4.27 %, 11 slices from eight mice; KO = 90.74 ± 4.5 %, 9 slices from eight mice; unpaired t-test, *p* < 0.03). **c**, Depotentiation in wild-type (WT = 131.55 ± 8.3 %, 8 slices from four mice) and *cAMP-GEF II* ^*−/−*^ mice (KO = 121.69 ± 9.7 %, 8 slices from four mice; arrow, three trains of theta-burst stimulation). **d**, There was no difference in depotentiation between wild-type and *cAMP-GEF II* ^*−/−*^ mice during the last 5 min of recording. Abbreviations: fEPSP, field excitatory postsynaptic potential. NS, no significance

Because cAMP-GEF II activates Rap1 as an alternative target of cAMP, and the NMDAR-Rap1-p38 MAPK pathway is involved in LTD [6, 11], we assessed NMDAR-LTD induced by low-frequency stimulation (LFS, 1 Hz for 15 min) at SC-CA1 synapses [12] of *cAMP-GEF II*^*−/−*^ mice. We found that NMDAR-LTD was absent in *cAMP-GEF II*^*−/−*^ mice (90.74 ± 4.51 % of baseline, n = 9 slices from eight animals; *p* < 0.03) compared to wild-type mice (75.45 ± 4.28 % of baseline, n = 11 slices from eight animals) (Fig. 3a, b). cAMP-GEFs can activate Rap2 as well as Rap1 [1, 2]. It has been reported that the Rap2-c-Jun N-terminal kinase (JNK) pathway is responsible for synaptic depotentiation, which is another form of synaptic depression [11]. Therefore, we assessed depotentiation in the hippocampal CA1 area and found that it was normal in SC-CA1 synapses in *cAMP-GEF II*^*−/−*^ mice (121.69 ± 9.71 % of baseline, n = 8 slices from four animals) compared to wild-type mice (131.55 ± 8.31 % of baseline, n = 8 slices from four animals) (Fig. 3c, d), suggesting that cAMP-GEF II deficiency did not affect depotentiation through Rap2. Taken together, our findings imply that cAMP-GEF II is important for NMDAR-LTD induction at hippocampal SC-CA1 synapses.

### Behavioral flexibility is altered in *cAMP-GEF II*^*−/−*^ mice

**Fig. 4:**
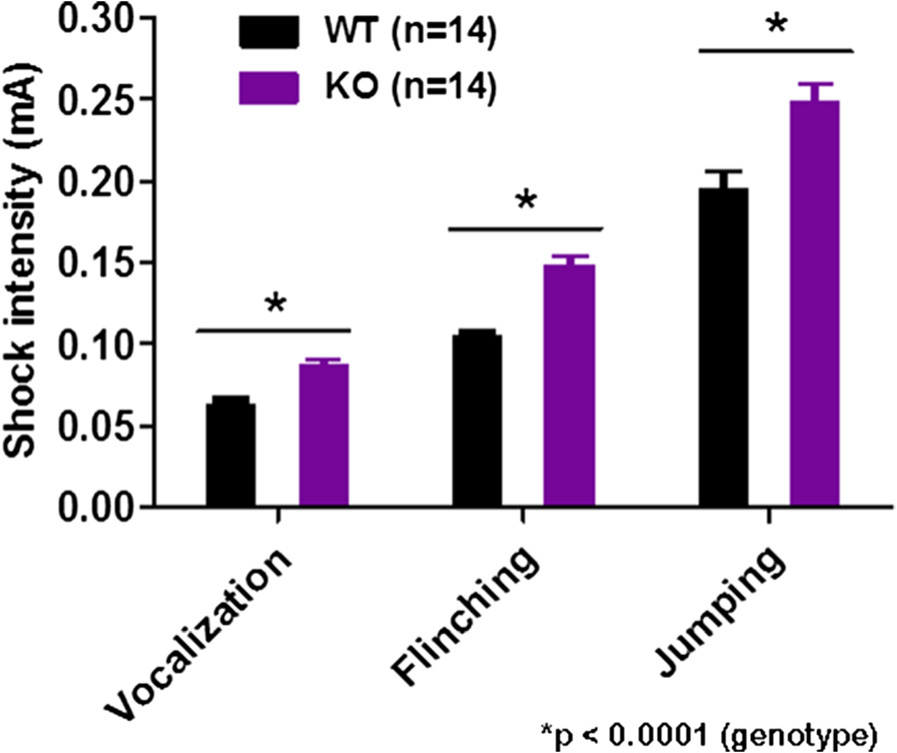
Foot shock sensitivity test in 12-month-old male mice. KO mice showed significantly less sensitivity to foot shock stimuli (12-month-old male mice; n = 14 mice per genotype; Two-way ANOVA; F_(1, 83)_ = 27.03, *p < 0.0001 for genotype; F_(2,83)_ = 64.0, *p* < 0.00001 for behavior; F_(2,83)_ = 1.14, *p* = 0.32 for genotype and behavior). Data are shown as mean ± SEM

**Fig. 5:**
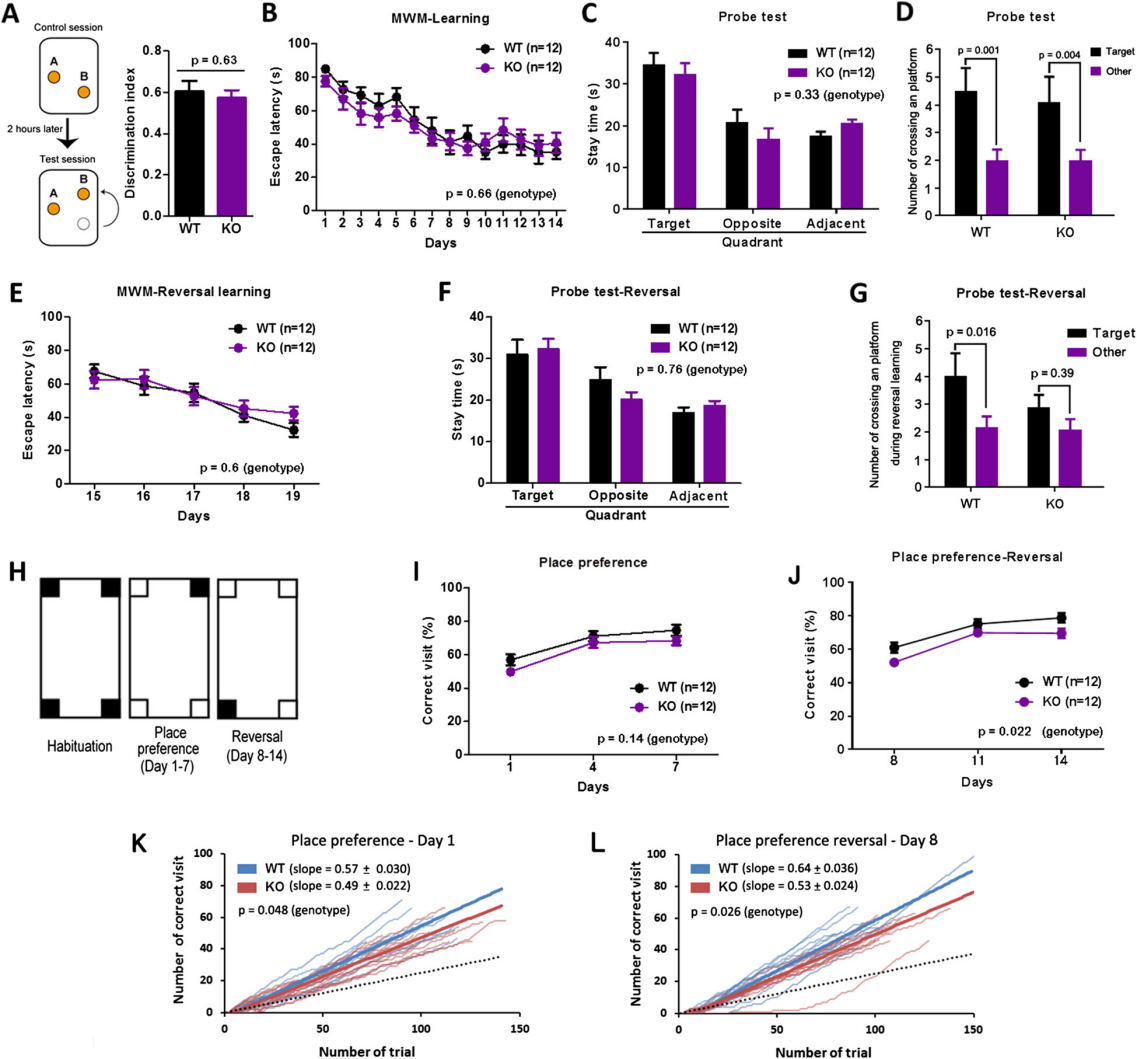
*cAMP-GEF II* ^*−/−*^ mice showed impaired reversal learning in the place preference learning task. **a**, Novel location recognition test. Left panel, experimental design. Right panel, no difference between genotypes in the discrimination index, which indicates that spatial memory is normal in *cAMP-GEF II* ^*−/−*^ mice (6-month-old male mice; wild-type (WT) = 10 mice; *cAMP-GEF II* ^*−/−*^ (KO) = 12 mice; unpaired t-test, *p* = 0.63). The discrimination index was calculated as follows: discrimination index = (contact duration for object B)/(total contact duration for objects). **b**, There were no differences in escape latency between genotypes in the Morris water maze test during training days from day 1 to 13 (6-month-old male mice; 12 mice per genotype; Two-way RM ANOVA, F_(1, 22)_ = 0.20, *p* = 0.66 for genotype; F_(13,286)_ = 11.28, *p* < 0.00001 for day; F_(13,286)_ = 0.60, *p* = 0.85 for genotype and day interaction). **c**, Stay time (WT = 34.54 ± 2.85 s; KO = 32.25 ± 2.69 s) in the initial target quadrant during a probe trial on day 14 showed that *cAMP-GEF II* ^*−/−*^ mice have similar spatial memory to wild-type mice (Two-way ANOVA; F_(1, 66)_ = 0.94, *p* = 0.33 for genotype; F_(2,66)_ = 17.76, *p* < 0.00001 for quadrant; F_(2,66)_ = 0.41, *p* = 0.66 for genotype and quadrant interaction). **d**, Wild-type and *cAMP-GEF II* ^*−/−*^ mice crossed more frequently the platform position in the target quadrant where the platform was located than pseudo-positions in other quadrants (Two-way ANOVA; F_(2,90)_ = 19.71, *p* < 0.00001 for position; F_(1,90)_ = 0.25, *p* = 0.62 for genotype; F_(2,90)_ = 1.28, *p* = 0.28 for genotype and position interaction; post-hoc Bonferroni test *p* = 0.001 between positions in WT and *p* = 0.004 between positions in KO) during a probe trial after initial learning. **e**, Escape latency to the new platform during reversal training was not different between genotypes (two-way RM ANOVA; F_(1, 22)_ = 0.27, *p* = 0.61 for genotype; F(4,88) = 14.92, p < 0.00001 for day; F_(4,88)_ = 0.95, *p* = 0.44 for genotype and day interaction). **f**, Stay time in the new target quadrant during a reversal probe trial on day 19. Wild-type and *cAMP-GEF II* ^*−/−*^ mice showed significant preference for the new target quadrant compared to opposite or adjacent quadrants, resulting in no difference between genotypes (Two-way ANOVA; F_(1, 90)_ = 0.1, *p* = 0.75 for genotype; F_(2,90)_ = 21.84, *p* < 0.00001 for quadrant; F_(2,90)_ = 1.27, *p* = 0.29 for genotype and quadrant interaction). **g,** *cAMP-GEF II* ^*−/−*^ mice crossed less frequently the platform position in the new target quadrant during the reversal probe trial (Two-way ANOVA; F_(1,92)_ = 5.48, *p* = 0.021 for position; F_(1,92)_ = 1.5, *p* = 0.22 for genotype; F_(1,92)_ = 1.24, *p* = 0.27 for genotype and position interaction; post-hoc Bonferroni test *p* = 0.015 between positions in WT and *p* = 0.39 between positions in KO). **h**, Experimental scheme for place preference and reversal learning test in IntelliCage. Performance was quantified as the percentage of correct corner visits (4-month-old female mice; 12 mice per genotype). **i**, There was no difference in spatial memory between the two genotypes in the place preference learning test (Two-way RM ANOVA; F_(1, 22)_ = 2.35, *p* = 0.14 for genotype; F_(2,44)_ = 71.2, *p* < 0.00001 for day; F_(2,44)_ = 0.55, *p* = 0.58 for genotype and day interaction). **j**, The percentage of correct corner visits in the reversal learning test was significantly reduced in *cAMP-GEF II* ^*−/−*^ mice, indicating a deficit in behavioral flexibility (Two-way RM ANOVA; F_(1, 22)_ = 6.03, *p* = 0.022 for genotype; F_(2,44)_ = 64.0, *p* < 0.0001 for day; F_(2,44)_ = 0.84, *p* = 0.43 for genotype and day interaction; post-hoc unpaired t-test, day 8 ( *p* = 0.0298), day 11 (*p* = 0.1376), day 14 (*p* = 0.0306). **k** and **l**, Learning speeds in the first days of the place preference (**k**, day 1) and reversal learning (**l**, day 8) tests. The slope of the learning curve in each mouse was determined by the least squares analysis. Black dashed lines indicate the chance level. Thin and thick blue lines represent wild-type mice and average, respectively. Thin and thick red lines represent *cAMP-GEF II* ^*−/−*^ mice and average, respectively. The slope was significantly decreased in *cAMP-GEF II* ^*−/−*^ mice in both place preference (PP) and place preference reversal (PPR) tests (for PP: WT, slope = 0.57 ± 0.03; *cAMP-GEF II* ^*−/−*^, slope = 0.49 ± 0.022; Unpaired t-test, *p* = 0.048; for PPR, WT, slope = 0.64 ± 0.036; KO, slope = 0.53 ± 0.024; Unpaired t-test, *p* = 0.026). All data are shown as mean ± SEM

We examined whether deficits of hippocampal synaptic plasticity in the *cAMP-GEF II*^*−/−*^ mice are accompanied by alterations of hippocampal-dependent cognitive functions. Unfortunately, we could not use a contextual fear conditioning test because *cAMP-GEF II*^*−/−*^ mice were less sensitive to foot shock stimuli compared to wild-type mice (Fig. 4). Therefore, we used the novel location recognition test, Morris water maze task, and IntelliCage test [13, 14]. In the novel location recognition test, which is a simple assay for hippocampal-dependent spatial memory [15], we found that both genotypes of mice equally exhibited a preference for novel location of an identical object, which resulted in a similar discrimination index (Fig. 5a). In the Morris water maze task we also found that there was no difference in performance during training from day 1 to day 14 (Fig. 5b), or in a probe trial on day 14 (Fig. 5c, d) between wild-type and *cAMP-GEF II*^*−/−*^ mice, which indicates normal spatial learning and memory in *cAMP-GEF II*^*−/−*^ mice. We then tested reversal learning by changing the position of the hidden platform to the opposite of the initial quadrant in the pool, and found that both wild-type and *cAMP-GEF II*^*−/−*^ mice equally learned the new target position (Fig. 5e). Additionally, both genotypes of mice did not show any significant differences in the preference for the new target quadrant during the probe trial of reversal learning on day 19 (Fig. 5f). To evaluate the accuracy of spatial memory, we analyzed the crossing numbers of the platform position in both of the probe tests. We found no differences between genotypes in the first probe test after initial learning. However, *cAMP-GEF II*^*−/−*^ mice tended to cross the target platform position less frequently than wild-type mice in the second probe test after reversal learning (Fig. 5g), suggesting a low accuracy of reversal memory in *cAMP-GEF II*^*−/−*^ mice. As mentioned above, since *cAMP-GEF II*^*−/−*^ mice showed less sensitivity to painful or aversive stimuli such as foot shock compared to wild-type mice, we wanted to confirm hippocampal cognitive functions using preference behavior rather than avoidance behavior in *cAMP-GEF II*^*−/−*^ mice. In contrast to the Morris water maze task, which is based on the avoidance behavior of mice escaping from water, the automated IntelliCage apparatus enabled us to measure spatial memory and reversal learning using preference behavior to reward, without the intervention of experimenters. Therefore, we performed the place preference learning test using the IntelliCage system for 7 days for spatial memory, and for the next 7 days for reversal learning (Fig. 5h). We found that initial spatial memory was normal (Fig. 5i), while reversal learning was significantly impaired (Fig. 5j) in *cAMP-GEF II*^*−/−*^ mice. We also measured the learning speeds of each mouse in the first days of the place preference and reversal learning tests. We found significant differences in the learning speeds between genotypes in both tests (Fig. 5k, l for place preference and reversal learning test, respectively). The difference was, however, more significant in the reversal learning test. These results indicate that *cAMP-GEF II−/−* mice showed slower learning than WT especially in the case of the reversal learning. Taken together, these findings from the Morris water maze and IntelliCage tests suggest that deletion of cAMP-GEF II may affect spatial learning and memory acquisition, and it may contribute to impairment of hippocampal-dependent reversal learning with a reduction in behavioral flexibility. These results are consistent with previous reports showing that hippocampal LTD is related to memory processes [16] and behavioral flexibility in spatial learning [17, 18].

## Discussion

In the present study, we examined the role of cAMP-GEF II in synaptic plasticity and hippocampus-dependent cognitive function using genetic approaches. We found that function of cAMP-GEF II is more closely related to NMDAR-LTD than to LTP or depotentiation, and that the alteration of synaptic responses and plasticity was associated with postsynaptic changes in the SC-CA1 pathway of the hippocampus. In addition, the impairment in NMDAR-LTD was accompanied by a reduction of behavioral flexibility in *cAMP-GEF II*^*−/−*^ mice.

### cAMP-GEF II in presynaptic axon terminals and postsynaptic densities

Modulation of presynaptic transmission and remodeling of postsynaptic spines are known to play a critical role in synaptic plasticity of brain circuits and in cognitive functions such as memory formation [19, 20]. In both pre- and postsynaptic processes, secondary messengers such as cAMP are key components regulating synaptic strength [21]. Previous studies on the effect of cAMP on synaptic plasticity have shown that PKA activation by cAMP is an essential step [22]. However, we cannot rule out the role of other cAMP-dependent (but PKA-independent) mechanisms such as the one involving cAMP-GEFs in synaptic function. Previous pharmacological studies using the cAMP-GEFs agonist 8-CPT-cAMP in *Drosophila* [23], exciter nerve axon of crayfish neuromuscular junction [24], calyx of Held of rat [25], and cultured hippocampal neurons [4], demonstrated that cAMP facilitates presynaptic transmission by increasing the number of neurotransmitter-releasing vesicles through activation of the PKA-independent cAMP-GEFs pathway in axon terminals. However, in the present study, and in agreement with a previous report by Yang and colleagues using Epac2 null mice [9], we could not find any evidence for a role of cAMP-GEF II in presynaptic transmission in the hippocampal SC-CA1 synapses of *cAMP-GEF II*^*−/−*^ mice. Therefore, we assume that discrepancies may arise from differences between animal species or type of neurons used for experiments, for instance exciter nerve axon of cray fish [24] or calyx of Held of rat [25] versus hippocampal CA1 pyramidal neuron in mice used in this study. It should be also noted that 8-CPT-cAMP activates both cAMP-GEF I and II. In any case, our data suggest that the impairment in hippocampal synaptic plasticity observed in *cAMP-GEF II*^*−/−*^ mice was induced by postsynaptic alterations, rather than presynaptic changes. Furthermore, western blot analysis using synaptic membrane fractions containing PSDs also supported the postsynaptic function of cAMP-GEF II. Supporting this, a proteomic study using mass spectrometry (LC-MS/MS) detected cAMP-GEF II protein in forebrain PSDs [26], and cAMP-GEF II protein colocalized with the postsynaptic marker PSD-95, suggesting a functional role for cAMP-GEF II in dendritic spines [20]. All these data strongly support our findings on the role of cAMP-GEF II in postsynaptic function.

### cAMP-GEF II and long-term depression

It has been previously shown that the small GTPase Rap1 mediates NMDA receptor-dependent AMPA receptor internalization during LTD [6]. Moreover, Ster and colleagues [27] reported that in mouse hippocampal slices, cAMP-GEFs activation by 8-CPT-cAMP induced LTD with a postsynaptic mechanism dependent on the interaction of AMPA receptor and PDZ proteins, activation of small GTPase Rap1-p38 MAPK signaling, and intracellular Ca^2+^ stores. In agreement with this previous report, we show in our study that cAMP-GEF II is highly associated with LTD induction.

However, in contrast to the lack of LTD and behavioral flexibility shown in *cAMP-GEF II*^*−/−*^ mice in our study, a recent work has shown that cAMP-GEF I/II double-null mice on a 129Sv background presented impairment of LTP, but not LTD, with deficits in hippocampal spatial learning [9]. In addition, cAMP-GEF II specific knockout mice showed normal hippocampal synaptic function and memory [9]. The discrepancies between the data of Yang and colleagues [9] and ours, in both synaptic plasticity and behavioral testing, could be related to differences in the genetic background mice strains used. We used C57BL/6J mice, while Yang and colleagues [9] used 129Sv mice. Although inbred mouse strains are a powerful tool for a better understanding of gene function, brain region- and strain-specific variations in gene expression may yield differences in neural functions or neurobehavioral phenotypes across mouse strains [28, 29]. Indeed, several mouse genetic studies performed to assess mechanisms underlying neurobehavioral differences, detected that many genes were differentially expressed between C57BL/6 and 129Sv mouse strains [30, 31], and defined C57BL/6 and 129Sv mouse strains as different based on microarray gene expression profiling [32]. Although a differential expression of cAMP-GEF I or II between these two strains has not been reported, many genes related to signaling pathways such as Ras-GTPase activating protein and Ras-like protein expressed in neurons presented clear differences in gene expression in nervous tissue [30, 31]. Therefore, we cannot rule out the effect of strain differences in gene expression on neural features and behavioral phenotypes. Alternatively or additionally, allele differences may in part account for these discrepancies. For instance, there is a general concern on *cis*-effects of a selection marker gene cassette near the targeted locus for the phenotypes of knockout or knockin mice [33–35].

### cAMP-GEF II and behavioral flexibility

A large body of evidence has demonstrated that hippocampal synaptic depression plays an important role in memory processes [16, 36–38] and behavioral flexibility [17, 18]. In our study, we found that mice lacking cAMP-GEF II had a mild reduction in behavioral flexibility in the Morris water maze and a place preference learning task using the IntelliCage test. These results are consistent with impairment of hippocampal LTD in *cAMP-GEF II*^*−/−*^ mice, although we cannot simply conclude that the behavioral results were a consequence of LTD impairment. In fact, cAMP-GEF II seems to have various roles in hippocampal-dependent memory with different downstream signaling pathways. Ostroveanu and colleagues [7] reported that an intra-hippocampal injection of 8-CPT-cAMP enhanced memory retrieval in the contextual fear conditioning via the Rap1-p42/p44 MAPK (ERK 1/2) signaling pathway, while memory acquisition was not affected. These results indicate that change in cAMP-GEF II activity is related to a variety of synaptic processes and cognitive functions, including behavioral flexibility and memory retrieval with distinct signaling pathways.

## Conclusions

In our study, we verified a specific role of cAMP-GEF II in NMDAR-LTD induction and behavioral flexibility in hippocampal-dependent reversal learning, using a genetic deletion approach.

## Methods

### Generation of cAMP-GEF II^−/−^ mice

The cAMP-GEFII floxed (with PGK-neo) allele was generated inserting a loxP into the 0.5 kb upstream of exon 3 and a FRT-pgk-neo-FRT-loxP cassette into the 0.5 kb downstream of exon 3. This line was generated using MS12 ES cell lines derived from the C57BL/6 strain [39], and maintained in a C57BL/6J genetic background. The cAMP-GEF II knockout (KO, *cAMP-GEF II*^*−/−*^) allele was generated by inducing Cre-mediated recombination in the germline of cAMP-GEFII floxed mice.

All experiments were performed in accordance with RIKEN (Japan), Kyungpook National University (Korea), Seoul National University (Korea) regulations to minimize pain and discomfort to animals. All animal protocols were also in accordance with the guidelines for the Care and Use of Laboratory Animals of the National Institutes of Health (NIH, USA).

### PCR for genotyping

PCR primer pairs (Fig. 1a) for genotyping were as follows: P1, 5′-GTGTTACTCTAGAAACGAC-3′/ P2, 5′-TGTTTCGCCAAGGGGATATTG-3′/P3, 5′-CTGGTGCTCACACCTCGTAC-3′ (630- and 250-bp bands for wild-type and *cAMP-GEF II*^*−/−*^ alleles, respectively).

### Western blot analysis

Western blots were performed as previously reported [40, 41] with some modifications. In brief, cortex tissues were dissected out immediately after cervical dislocation. Tissues were homogenized on ice in 10-volume buffer A (5 mM HEPES, pH 7.4 containing 0.32 M sucrose) containing a protease inhibitor (Roche, cat# 04693159001) and PhosSTOP (Roche, cat# 04906845001) using a Teflon homogenizer. Samples were centrifuged at 1,400 × *g* for 5 min at 4 °C, and the resulting supernatants (S1) were further centrifuged at 14,200 × *g* for 20 min at 4 °C. Pellets (P2, crude membrane fraction) were suspended and lysed in 6 mM Tris buffer (pH 8.0, containing 0.5 % Triton X-100) on ice for 30 min. The SPM was fractionated using a layered sucrose gradient (0.8 M, 1.0 M, and 1.2 M sucrose in 5 mM HEPES) at 82,700 × *g*. The interface between 1.0 M and 1.2 M sucrose was retrieved, which included postsynaptic membranes and PSD proteins without presynaptic vesicles. Proteins of S1, P2, and SPM were separated using SDS-polyacrylamide gel electrophoresis, and electroblotted to polyvinylidene fluoride (PVDF) membranes. Membranes were immunoreacted with an anti-cAMP-GEF II polyclonal antibody (diluted 1:1000; Santa Cruz, cat# SC-25633), anti-Actin monoclonal antibody (1:10,000; Millipore, Cat# MAB1501), or anti-PSD95 polyclonal antibody (1:5000; Frontier Institute, Cat# PSD95-GP-Af248-2), and their appropriate species-specific HRP-conjugated secondary antibodies. Finally, immunoreactive bands were detected using Luminata Forte Western HRP Substrate (Millipore, cat# WBLUF0500).

### Hippocampal slice preparation

Hippocampal slices were prepared from 3- to 5-week-old wild-type and *cAMP-GEF II*^*−/−*^ mice (male and female). For depotentiation experiment 10- to 12-week-old animal was used to differentiate its effect with LTD. Animals were anesthetized with 2-bromo-2-chloro-1,1,1-trifluroethane and decapitated. Brains were then removed and placed in ice-cold artificial cerebrospinal fluid (ACSF), which was aerated with 95 % O_2_ and 5 % CO_2_. The ACSF contained the following: 124 mM NaCl, 2.5 mM KCl, 1 mM NaH_2_PO_4_, 25 mM NaHCO_3_, 10 mM glucose, 2 mM CaCl_2_, and 2 mM MgSO_4._ Transverse hippocampal slices (400-μm thick) were prepared using a manual tissue chopper (MK-MTC9100, Mickle Laboratory Engineering) and allowed to recover in ACSF at room temperature for 1 h. After preparation, slices were transferred to a recording chamber maintained at 28 °C, and then continuously perfused with aerated ASCF at a rate of 1.5 mL/min, before recordings were obtained.

### Electrophysiological recordings

Extracellular recordings were performed in the *stratum radiatum* of the CA1 area of hippocampal slices using a glass pipette filled with ACSF (1 MΩ) in order to measure the slope of evoked field excitatory postsynaptic potentials (fEPSPs). Schaffer collateral fibers were stimulated every 30 s using bipolar electrodes (MCE-100, Kopf Instruments). fEPSPs were amplified using an Axopatch 200B amplifier, and digitized with a Digidata 1322A A/D board for measurement, at a sampling rate of 10 kHz. Data were monitored and analyzed using the WinLTP program [42]. Each experiment was conducted on separate slices, thus the n number represents the number of slices used for the experiment. For LTP and LTD, the stimulation intensity was adjusted to obtain fEPSP slopes of 45 % of the maximum. After a stable baseline period of over 30 min, high frequency stimulation (a single train of tetanus, 100 Hz for 1 s) or low frequency stimulation (1 Hz for 15 min) were applied, respectively. Depotentiation was induced using three trains of theta-burst stimulation (consisting of five pulses at 100 Hz, and repeated five times at 5 Hz) at 10 s of intertrain interval, followed 30 min later by low frequency stimulation (2 Hz, 10 min). For PTP, the NMDA receptor antagonist D-APV (25 μM, Tocris) was added to the ACSF during recording. PPF was induced by stimulation of a pair of afferent fibers at short intervals (20, 50, 100, or 200 ms), which is sensitive to presynaptic release probability [10].

### Electrophysiology data analysis

Measurements were expressed as percentage of the averaged value calculated 10 min before LTP or LTD induction. Significant differences between groups were assessed using Student’s t-test of the last 10 min average values after LTP and last 5 min average values after LTD or depotentiation induction. Data are presented as mean ± SEM, and statistical significance was set at *p* < 0.05.

### Generation of Antibody

KLH-coupled synthetic peptides (CQMSHRLEPRRP) corresponding to the C-terminus of cAMP-GEFII were used to raise a rabbit polyclonal antibody (BSI Research Resources Center).

### Immunohistochemistry

Mice were fully anesthetized and a needle was inserted directly into the left ventricle. Animals were then perfused using 4 % paraformaldehyde pH 7.4 (0.5 mL/g of body weight) at a speed of 1 mL/min. Brains were removed, post-fixed in 4 % paraformaldehyde overnight at 4 °C, and cryoprotected in 0.1 M phosphate buffer (PB) containing 30 % sucrose. For immunohistochemistry of cAMP-GEF II, thin sections (5 μm thick) from paraffin-embedded samples were deparaffinized, rehydrated, and processed for heat-induced epitope retrieval. After blocking in 4 % normal goat serum for 1 h, tissue sections were reacted with a rabbit anti-cAMP-GEF II antibody (diluted 1:2000) at 4 °C overnight, and then incubated in biotinylated anti-rabbit IgG (1:200, Vector Laboratories, Burlingame, CA, USA) at room temperature for 2 h. After washing in phosphate buffered saline containing Triton X-100 (PBST), sections were incubated in avidin-biotin-peroxidase complex (1:250 dilution, ABC Elite; Vector Laboratories) at room temperature for 1 h. The horseradish peroxidase reaction was developed in 0.1 M Tris–HCl (pH 7.4) containing 0.05 % 3,3´-diaminobenzidine, and 0.01 % H_2_O_2_., and sections were dehydrated. Bright-field images were taken with a digital slide scanner (NanoZoomer; Hamamatsu Photonics). For immunofluorescence, tissue blocks were sectioned in the coronal plane (30 μm thick), and free-floating sections were post-fixed in 50 % ethanol for 10 min at room temperature. After blocking with 4 % normal goat serum, sections were permeabilized with 0.3 % Triton X-100 in phosphate buffered saline (PBS) for 3 h, and incubated with NeuN (1:1000 dilution, Millipore) antibody overnight at 4 °C. Immunolabeling was visualized using an anti-mouse secondary antibody conjugated to Alexa 488 (1:500, Invitrogen) at room temperature. Sections were then dehydrated, mounted on glass slides, and visualized using a confocal microscope (LSM700, Zeiss).

### Novel location recognition test

Mice (6-month-old males; n = 10 WT, n = 12 KO) were habituated to an empty cage (21 x 42 x 21 cm) for 10 min per day for 3 days before starting the experiment. For identical objects (A and B), two identical plant pots were used. On day 4, object A was placed in the center of the cage and object B was placed next to object A (i.e., control session). Mice were free to explore for 5 min in the cage, and then they were moved to a homecage. Two hours later, location of object B was changed, and mice were free to explore for 5 min in the cage again (i.e., test session). The time spent touching an object was recorded from a camera mounted overhead, and was manually counted. The discrimination index calculation formula was as follows: discrimination index = (contact duration of object B)/(total contact duration of objects).

### Morris water maze task

The Morris water maze test was performed according to the procedure described previously by Nishiyama and colleagues [43], with some modifications. The water pool used in the current experiment was 1.5 m in diameter and illuminated with 300 lux white fluorescent light at the maze-surface level. The pool temperature was kept at 25 ± 1 °C. The acrylic transparent platform (diameter 10 cm) was submerged 0.7 cm below the surface of water made opaque by adding nontoxic white paint. The location of the platform was fixed over a series of trials for each mouse. If the mouse located the platform within 90 s, the mouse was allowed to remain on it for 30 s. Mice that failed to find the platform within 90 s were manually guided to the platform and allowed to remain on it for 30 s. Mice were given four trials per day for 19 consecutive days in a spaced manner. The inter-trial intervals for individual mice were about 30–60 min. A different randomly selected starting point along the rim of the maze was used for each of the four trials. On day 15, the platform position was changed to the opposite side of the initial target quadrant, and mice relearned the new platform position. A probe trial and a reversal probe trial were performed on days 14 and 19, respectively, after the acquisition sessions. In the probe tests, the platform was removed from the tank, and each mouse was allowed to swim for 90 s. Movement of each mouse in the maze was recorded using a video camera and analyzed with NIH IMAGE WM 2.12 (O’Hara & Co.) software.

### Place preference learning task with IntelliCage

The IntelliCage apparatus and software (NewBehavior AG) have been described previously [13, 14], and we performed the IntelliCage test as previously reported [44], with some modifications. Radiofrequency identification transponders (Planet ID GmbH) were implanted subcutaneously in the dorsocervical region. During all adaptation phases and tasks, mice were fed *ad libitum*. Adaptation phase was 3 weeks. During the first week, all doors were open; mice were free to access all four corners, which had water bottles (i.e., free adaptation). During the second week, all doors were closed but could be opened once per visit with a nose-poke for 5 sec (i.e., nose-poke adaptation). During the third week, mice were adapted to a fixed drinking schedule (i.e., drinking session adaptation) with doors opening in response to nose-pokes between the hours of 21:00–24:00 only. In the place preference task, water was available in only one of the four corners (i.e., correct corner) during the drinking session. This task was performed for 7 days, and the number of corner visits was counted for 3 h. Performance was quantified as the percentage of correct corner visits. In the reversal learning task, water was available only in the opposite corner (i.e., new correct corner) during the drinking session. This task was also performed for 7 days, and the number of corner visits was counted for 3 h.

### Foot shock sensitivity test

Foot shock sensitivity was assessed by giving mice electrical shocks of increasing intensity, ranging from 0.05 mA to 1 mA, and monitoring their behavior (i.e., flinching, vocalization, and jump).

### Statistical analysis for behavioral tests

Data were analyzed using a Two-way ANOVA, Two-way repeated measurements ANOVA (RM ANOVA), and Unpaired t-tests. Probability values (*p*) less than 0.05 were considered statistically significant.
